# Visualizing Scholarly Trends in Stochastic Models for Disease Prediction

**DOI:** 10.7759/cureus.69033

**Published:** 2024-09-09

**Authors:** Sunila V, Jais Kurian, Liny Mariam Mathew, Pratheesh Mathew, Dary John, Jeena Joseph

**Affiliations:** 1 Research Department of Statistics, Nehru Arts and Science College Kanhangad, Kanhangad, IND; 2 Department of Mathematics, St Stephen's College Uzhavoor, Uzhavoor, IND; 3 Department of Mathematics, Devaswom Board College, Thalayolaparambu, IND; 4 Department of Mathematics, Nirmala College (Autonomous), Muvattupuzha, IND; 5 Department of Mathematics, Newman College, Thodupuzha, IND; 6 Department of Computer Applications, Marian College Kuttikkanam (Autonomous), Kuttikkanam, IND

**Keywords:** bibliometric analysis, biblioshiny, disease prediction, stochastic models, vosviewer

## Abstract

Stochastic models play a pivotal role in disease prediction by accounting for randomness and uncertainty in biological systems. This study offers a visualization of trends in the application of stochastic models for disease prediction from 1990 to 2024, based on a bibliometric analysis of Scopus data. Key findings reveal a significant growth in research post-2014, largely driven by global health challenges like COVID-19. Despite these advancements, gaps remain in applying these models to non-communicable diseases and low-resource settings. By integrating computational techniques like machine learning, stochastic models hold promise for improving predictive accuracy. This study highlights the need for further international collaboration and interdisciplinary research, offering practical insights for researchers and public health professionals aiming to enhance disease prediction and intervention strategies.

## Introduction and background

Stochastic models have become an important tool in the area of disease prediction because they allow one to take into account the underlying randomness and uncertainty involved in biological systems. In particular, they are very useful for studying the dynamics of spread, progression, and various interventions against diseases. In contrast to deterministic models based on a fixed outcome determined by the initial conditions, models allow for random variations and thus hold a better, more accurate representation of reality when demonstrating a disease process. The importance of this approach increases when modeling complex scenarios with high uncertainty, for example, the development of chronic diseases, infection dynamics, and even carcinogenesis [1]. The applications of stochastic models to the prediction of diseases are found to include cancer risk assessment, infectious disease modeling, and chronic disease forecasting, a versatile tool in epidemiological research [2,3].

Stochastic models have been applied to an extremely broad range of diseases, from cancer to infectious diseases, for the purpose of predicting outcomes. For instance, Lee and Zelen developed a stochastic model that predicts the breast cancer mortality rate with respect to advances in therapy and screening programs. Their model is based on the progressive transition between health states and has been instrumental in forecasting the impact of early diagnosis on mortality rates [4]. Similarly, Teng et al. presented a stochastic disease prediction model called StoCast for errors and uncertainty associated with the progression of diseases such as Alzheimer's and Parkinson's. This deep generative technique-based model provides robust predictions even in scenarios characterized by very high data uncertainty, enhancing decision-making in healthcare [5].

In the context of infectious diseases, Addy et al. developed the stochastic model of Hookfurther to include variable susceptibility and infectivity within a population, which was able to be successfully applied to influenza epidemics with the clustering pattern for infections in different age groups [6]. Further, Sene proposed a stochastic model that considers stochastic perturbations in order to simulate the spread of the novel coronavirus and provide insight into the rapid dissemination and uncertainty in the dynamics of diseases [2]. Stochastic models have been applied even in the study of the immunological response dynamics against viral infection. Yuan and Allen constructed stochastic models of HIV-1 infection that had the interesting derived result of there being a positive probability of extinction of the virus. This cannot be captured by any deterministic model. Their work pointed out the necessity of including random events in the dynamics of diseases, more so in connection with the immune system [7]. These studies further underscore both the power and significance of the stochastic model in deriving disease outcomes and giving an insight that otherwise might have been overlooked by purely deterministic models. Accordingly, research into stochastic modeling remains key to the advancement of pursuits in enhanced disease prediction and intervention strategies.

Bibliometric analysis, therefore, is one of the most powerful quantitative tools through which the evaluation and analysis of academia in any subject can be done by examining tendencies in research conducted in any field [8-12]. It is a systematic assessment of publications, citations, and other scholarly outputs with the purpose of discovering patterns and getting influential works that give intellectual structure to a discipline [13-16]. This form of analysis is performed using instruments such as Biblioshiny and VOSviewer. One of the tools is Biblioshiny, a web-based user-friendly interface for the R-based bibliometric package, which allows broad analyses and visualizations to be conducted by researchers who do not have extensive knowledge in programming [17-19]. Another tool is VOSviewer, which has been well utilized in generating and exploring bibliometric networks about co-authorship, co-citation, and keyword co-occurrence. These types of networks provide new insights into research by graphically representing complicated landscapes [20-21].

This study seeks to answer the following research question: How have stochastic models for disease prediction evolved over the past three decades, and what are the key trends, contributors, and gaps in this field, particularly in relation to low-resource settings and non-communicable diseases?

To address this question, we provide a comprehensive bibliometric analysis of research trends in the application of stochastic models for disease prediction from 1990 to 2024. By analyzing data from Scopus and using tools such as Biblioshiny and VOSviewer, we aim to identify influential contributors, emerging research themes, and trends in the integration of advanced computational techniques like machine learning. Additionally, the study will highlight gaps in the application of stochastic models in low-resource settings and for non-communicable diseases, emphasizing the need for international collaboration to advance this field. Through these insights, the study hopes to guide future research efforts, inform public health strategies, and enhance the use of stochastic models in disease prediction.

## Review

Materials and methods

For this study, Scopus was chosen as the primary source of bibliographic data due to its extensive range of high-quality journals and broader coverage of scientific fields compared to other databases. While Scopus provides a comprehensive overview of relevant literature, a potential limitation is its exclusion of certain specialized or regional databases, which could affect the inclusion of some publications or niche topics. Thus, future studies may benefit from comparing results across multiple databases. The publications were retrieved using the keywords "stochastic model," "disease," and "prediction," with no language restrictions applied. We focused exclusively on journal articles and conference papers, as these represent the primary sources of peer-reviewed scientific knowledge. The timeframe of 1990-2024 was selected to capture developments in stochastic modeling over the past three decades, which coincides with the increasing integration of computational methods into disease prediction models. Although earlier works might exist, 1990 was chosen as a starting point to ensure the relevance of the dataset.

The initial search yielded 709 documents from 373 sources, and the Preferred Reporting Items for Systematic Reviews and Meta-Analyses (PRISMA) methodology was employed for systematic selection. The process involved three key steps. First, data were extracted from Scopus using the specified keywords. Second, reviews, editorials, book chapters, letters, notes, and short surveys were excluded to focus on original research articles and conference papers. Additionally, studies not directly relevant to disease prediction, despite including "stochastic model" in the keywords, were removed after screening. Duplicates were removed through automated tools in Biblioshiny (K-Synth Srl, Naples, Italy), and articles missing essential metadata or citation details were excluded to maintain accuracy. The final dataset was saved as a CSV file, and subsequent bibliometric analysis was conducted using VOSviewer (Centre for Science and Technology Studies, Leiden University, the Netherlands) and Biblioshiny software, which facilitated the visualization of co-authorship networks, keyword co-occurrence maps, and citation networks to explore the intellectual structure of the field.

**Figure 1:**
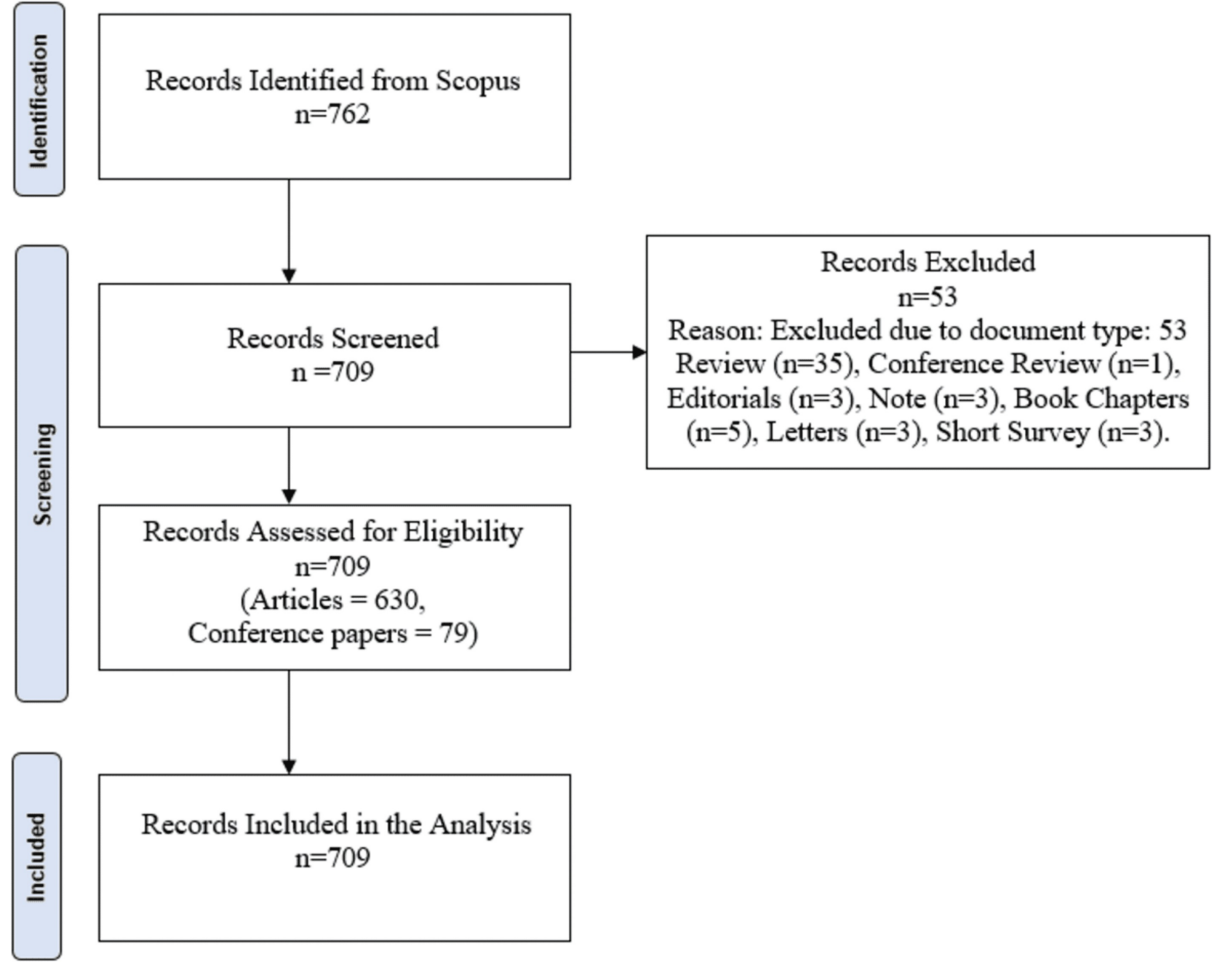
PRISMA approach PRISMA: Preferred Reporting Items for Systematic Reviews and Meta-Analyses (PRISMA)

Table 1 presents the key findings from the bibliometric analysis of stochastic models in disease prediction. The analysis spans from 1990 to 2024, covering 373 sources, including journals and books, and encompassing 709 documents. The field has exhibited an annual growth rate of 12.63%, with an average document age of 7.09 years and an average of 29.13 citations per document. A total of 29,168 references were cited across these documents. In terms of content, 7,395 keywords plus (ID) and 1,948 author's keywords (DE) were identified. The analysis also highlights the contributions of 3,098 authors, with 35 authors publishing single-authored documents and 39 single-authored documents in total. Collaboration is evident with an average of 4.93 co-authors per document and 33.15% of documents involving international co-authorships. Regarding document types, the analysis includes 630 articles and 79 conference papers.

**Table 1:** Main information of the investigation

Description	Results
Main information about data	
Timespan	1990-2024
Sources (journals, books, etc.)	373
Documents	709
Annual growth rate %	12.63
Document average age	7.09
Average citations per doc	29.13
References	29168
Document contents	
Keywords plus (ID)	7395
Author's keywords (DE)	1948
Authors	
Authors	3098
Authors of single-authored docs	35
Authors collaboration	
Single-authored docs	39
Co-authors per doc	4.93
International co-authorships %	33.15
Document types	
Article	630
Conference paper	79

Annual Scientific Production

**Figure 2:**
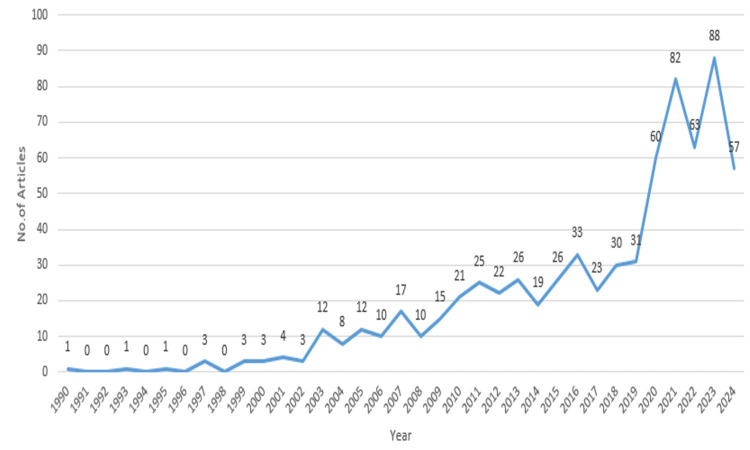
Annual scientific production The X-axis shows the years from 1990 to 2024. The Y-axis indicates the number of articles published in each corresponding year. Each data point on the line in the chart is annotated with the corresponding number of articles published that year.

Figure 2 illustrates the annual scientific production in the field of stochastic models applied to disease prediction from 1990 to 2024. The data shows a general upward trend in publications, with a significant increase beginning around 2014. Prior to this, scientific output remained relatively low and stable, with minor fluctuations. Post-2014, there was a marked rise in publications, peaking in 2022, which reflects growing interest and rapid advancements in the field during this period. The slight decline observed after 2022 warrants further exploration. Several factors may contribute to this drop in output. It could be a temporary fluctuation resulting from the completion of major research projects or shifts in funding priorities. Additionally, the COVID-19 pandemic may have impacted research resources and focus, as many researchers redirected efforts toward pandemic-related studies. Alternatively, the decline could indicate a stabilization in research output as the field matures, or it may reflect an evolving research landscape where newer approaches, such as hybrid models or interdisciplinary methods, are gaining traction. Given the recent nature of this decline, it is difficult to definitively classify it as a temporary fluctuation or a longer-term trend. Continued monitoring of publication trends in the coming years will provide greater insight into the future trajectory of research in stochastic models for disease prediction.

Most Relevant Authors

Table 2 lists the most relevant authors contributing to the field of stochastic models in disease prediction. Wang Y leads with 13 articles, making a significant impact in this area of research. Following are Wang X with eight articles and several authors, including Gilligan CA, Keeling MJ, Tildesley MJ, and Zhang Y, each with six articles. Other key contributors are Cao J, Grenfell BT, Li Y, and Liu Y, each authoring five articles. These authors represent some of the most influential voices in the field, contributing to the advancement and application of stochastic modeling techniques in disease prediction. For instance, Wang Y has contributed through the development of models like the Stochastic Disease Forecasting Model, which improves forecasting accuracy by dealing with uncertainties in clinical data for certain diseases. Keeling MJ has been influential in advancing methods for studying stochastic dynamics in epidemiology, showing how stochastic simulation can provide deep insights into disease extinction and control measures. These authors' work has provided significant theoretical and practical contributions, enhancing disease control measures and advancing stochastic modeling methodologies.

**Table 2:** Most relevant authors

Authors	Number of articles
Wang Y	13
Wang X	8
Gilligan CA	6
Keeling MJ	6
Tildesley MJ	6
Zhang Y	6
Cao J	5
Grenfell BT	5
Li Y	5
Liu Y	5

Most Relevant Sources

Table 3 presents the most relevant sources for publications on stochastic models in disease prediction. The leading source is PLOS Computational Biology with 48 articles, followed closely by PLOS ONE with 41 articles. Journal of Theoretical Biology ranks third with 22 articles, while Mathematical Biosciences and Journal of the Royal Society Interface contribute 16 and 14 articles, respectively. Other notable sources include Epidemics with 10 articles, Proceedings of the National Academy of Sciences of the United States of America with 10 articles, Infectious Disease Modelling with nine articles, and both Chaos, Solitons and Fractals and Computers in Biology and Medicine with eight articles each. These journals represent the core outlets for research on stochastic modeling in disease prediction, highlighting the interdisciplinary nature and broad relevance of this field across computational biology, theoretical biology, and applied mathematics.

**Table 3:** Most relevant sources

Sources	Number of articles
Plos Computational Biology	48
Plos One	41
Journal of Theoretical Biology	22
Mathematical Biosciences	16
Journal of the Royal Society Interface	14
Epidemics	10
Proceedings of the National Academy of Sciences of the United States of America	10
Infectious Disease Modelling	9
Chaos, Solitons and Fractals	8
Computers in Biology and Medicine	8

Trend Topics

**Figure 3:**
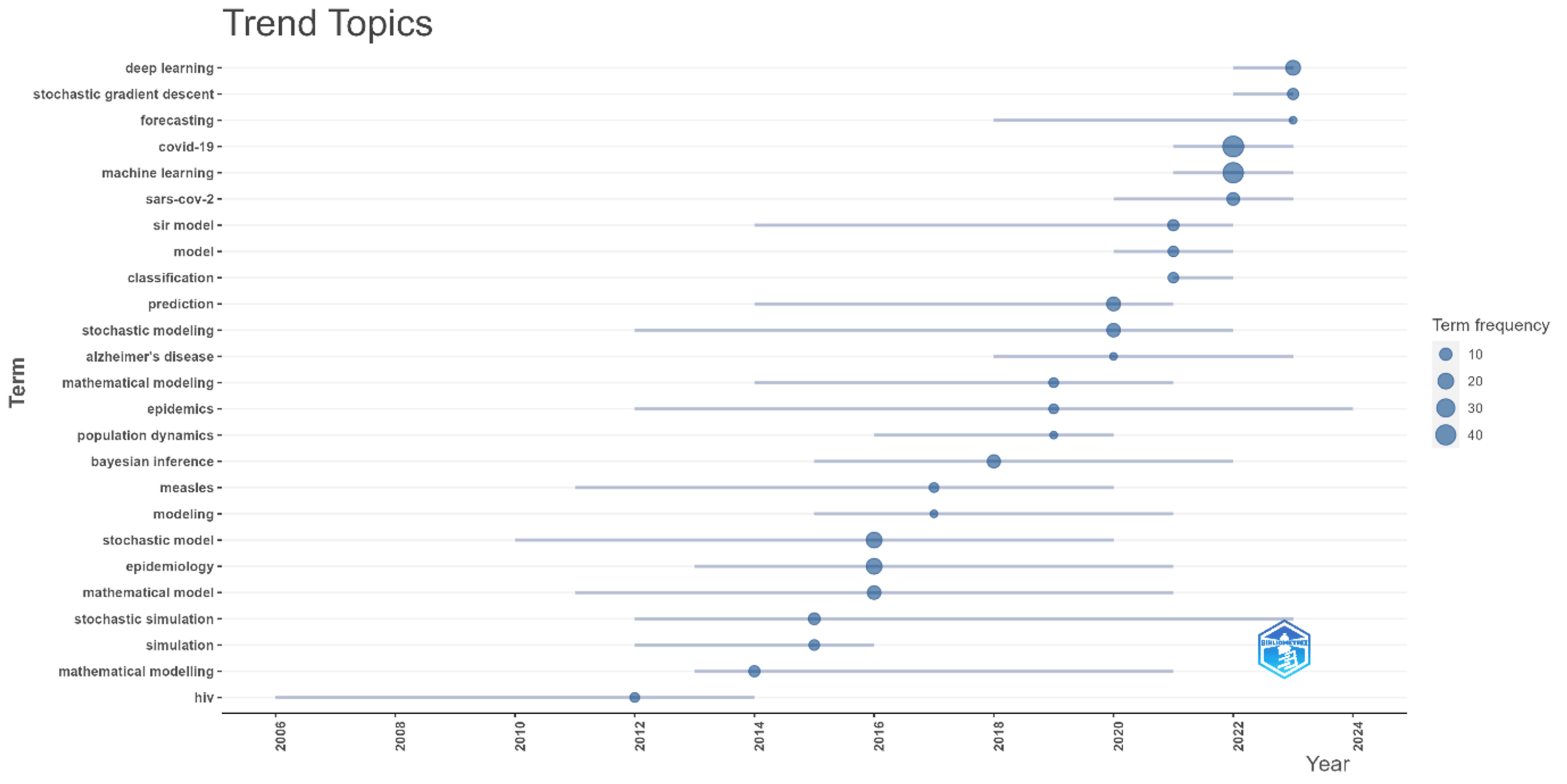
Trend topics

Figure 3 illustrates the trend topics in stochastic modeling applied to disease prediction over time. The graph showcases the emergence and frequency of key terms from 2006 to 2024. Early in the timeline, terms like "HIV," "stochastic model," "modeling," and "mathematical modeling" were prominent, reflecting the initial focus areas in this field. As the years progressed, the emergence of new topics such as "epidemics," "Bayesian inference," "machine learning," and "COVID-19" indicates a shift toward more complex and contemporary challenges in disease prediction. Recent years, particularly post-2020, show a strong emphasis on terms like "deep learning," "stochastic gradient descent," "SARS-CoV-2," and "forecasting," which align with the growing intersection of AI techniques and pandemic-related research. The size of the bubbles represents the frequency of these terms, with larger bubbles indicating higher relevance or usage within the literature. This visualization highlights how the focus of research in stochastic disease modeling has evolved, with a clear trend toward integrating advanced computational methods and addressing urgent global health issues.

Thematic Map

**Figure 4:**
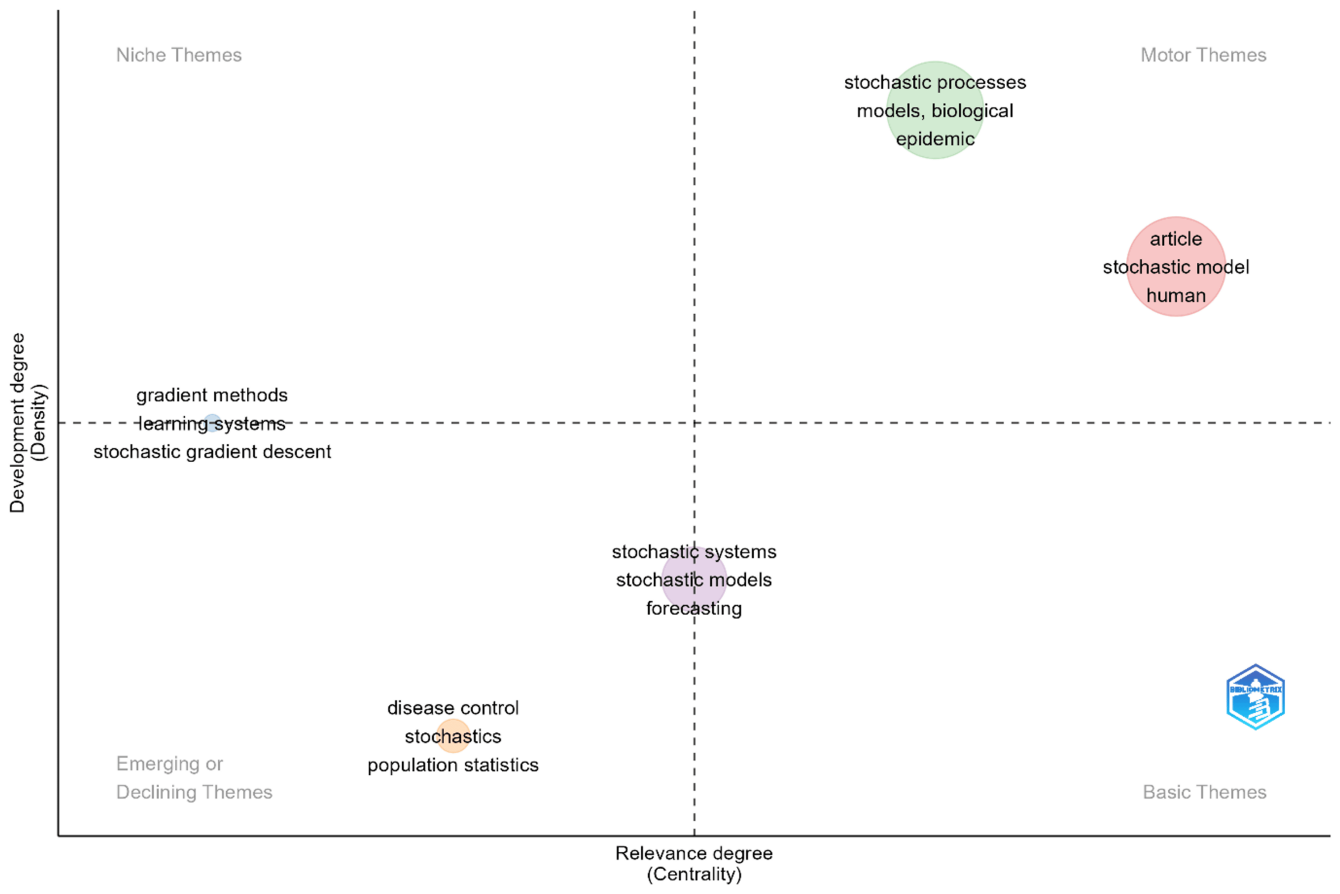
Thematic map

Figure 4 presents a thematic map of research topics related to stochastic models in disease prediction, categorizing them by their relevance (centrality) and development (density). The top-right quadrant, labeled Motor Themes, includes topics such as "stochastic processes," "biological models," and "epidemic." These themes are highly central and well-developed, suggesting that they are key to the field’s progression and are likely to continue driving significant research advancements. In the top-left quadrant, Niche Themes like "gradient methods" and "stochastic gradient descent" are well-developed but more specialized. These topics are less central to the broader field but represent areas of increasing computational importance, especially as stochastic models are refined with advanced computational techniques. The bottom-right quadrant contains Basic Themes such as "stochastic systems," "stochastic models," and "forecasting." These fundamental areas provide the groundwork for ongoing research, though they are less developed compared to Motor Themes. As computational capabilities grow, these foundational topics are expected to evolve, providing a basis for future advancements in disease prediction models.

The bottom-left quadrant, labeled Emerging or Declining Themes, includes topics such as "disease control," "stochastics," and "population statistics." While some of these themes appear to be declining in prominence, this may not necessarily indicate a reduction in research interest. Instead, certain traditional themes, such as "stochastics" and "population statistics," may be absorbed into emerging areas like machine learning and deep learning, which offer more sophisticated approaches for handling large datasets and complex systems. These emerging fields influence the evolution of traditional stochastic modeling by enhancing predictive accuracy and scalability in health scenarios, particularly in complex diseases like cancer and pandemics. The integration of deep learning techniques into stochastic models has led to more refined hybrid approaches that can manage high-dimensional data and provide real-time predictions. For instance, deep learning’s ability to process large datasets complements the randomness inherent in stochastic models, enhancing their ability to predict disease progression in uncertain environments. As a result, while some themes may seem to be declining, they are likely being integrated into newer interdisciplinary approaches rather than disappearing altogether. This thematic map provides a comprehensive overview of the current state of research and highlights potential future directions. It underscores the influence of both well-established and emerging topics, offering insights into how the field of stochastic modeling is evolving, particularly with the incorporation of machine learning and deep learning techniques.

Three Field Plot

**Figure 5:**
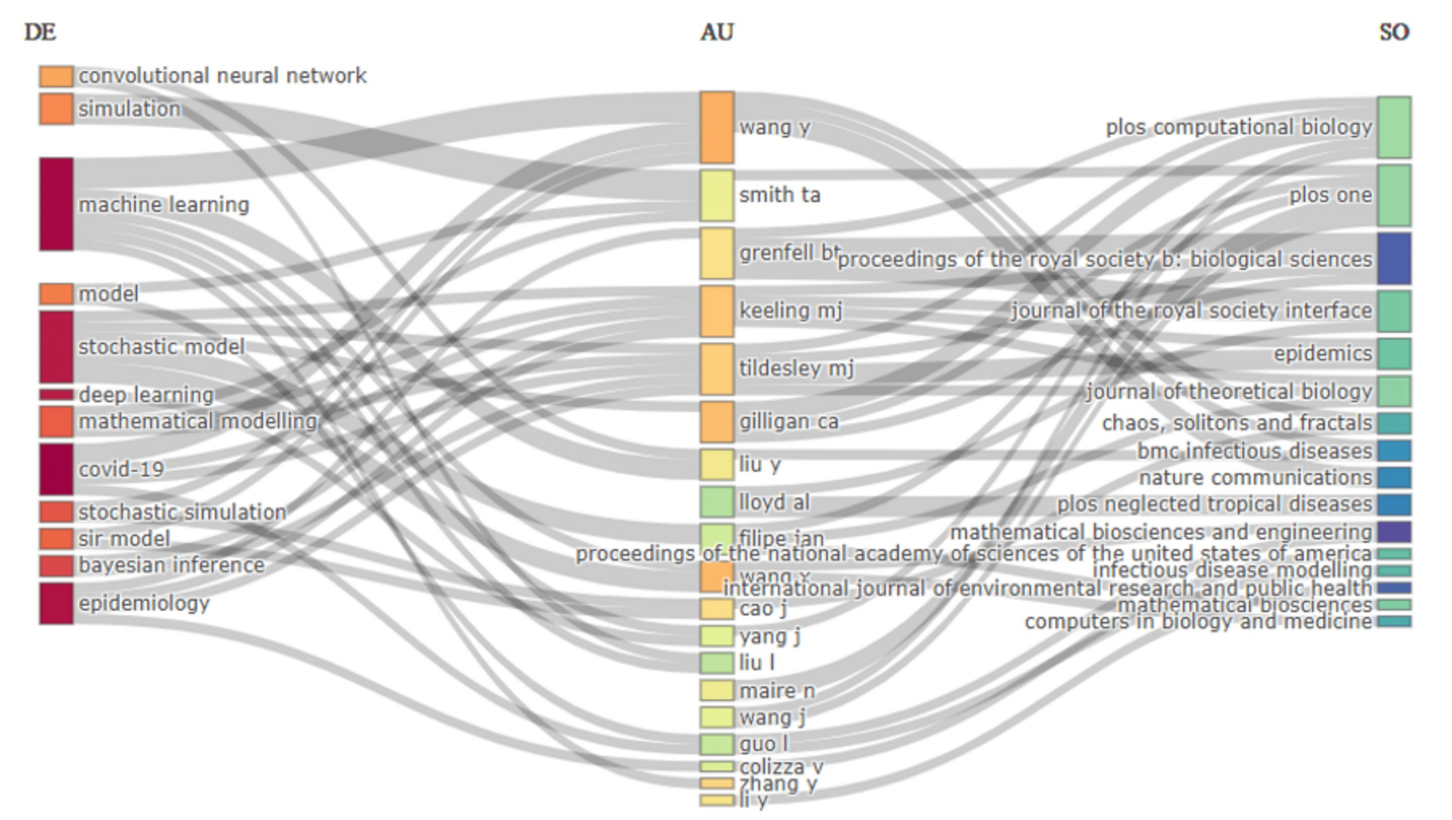
Three-field plot showing the connections between keywords, authors, and publication sources in stochastic models for disease prediction research

Figure 5 presents a three-field plot that visualizes the connections between author keywords (left), authors (middle), and sources (right) in the research on stochastic models for disease prediction. The plot highlights how specific keywords are associated with certain authors and how these authors' work is distributed across various journals. For instance, terms like "machine learning," "stochastic model," and "deep learning" are strongly connected to prolific authors such as Wang Y, Keeling MJ, and Tildesley MJ, whose research is published in leading journals like PLOS Computational Biology, Journal of Theoretical Biology, and Epidemics. The plot also shows that some authors, such as Smith TA and Grenfell BT, have a diverse set of keywords linked to their work, indicating their involvement in multiple aspects of stochastic modeling and disease prediction. Journals like PLOS ONE and Proceedings of the Royal Society B: Biological Sciences serve as key publication venues for these studies, showcasing a broad dissemination of research across interdisciplinary platforms. This visualization effectively demonstrates the interrelationships among key concepts, influential researchers, and prominent publication outlets within the field.

Co-authorship Between Countries

**Figure 6:**
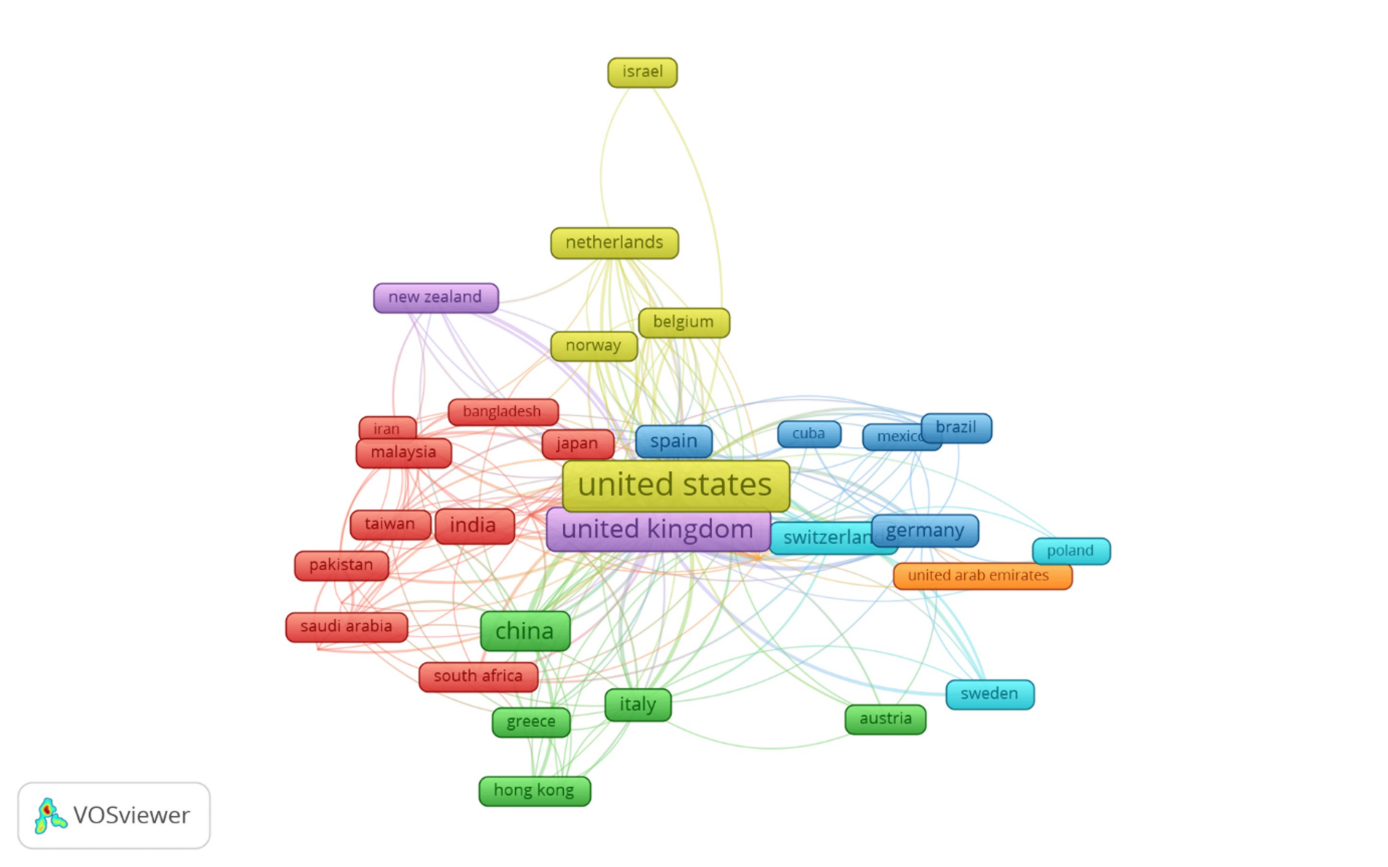
Co-authorship between countries

Figure 6 illustrates the co-authorship network between countries involved in research on stochastic models for disease prediction. The size of the nodes represents the number of publications co-authored by researchers from each country, while the thickness of the connecting lines indicates the strength of the collaborative ties between them. The network reveals several key insights into the global distribution and collaboration patterns in this research field.

The United States and the United Kingdom emerge as the most prominent hubs in the network, with substantial co-authorship connections to a wide range of countries. This indicates that these two countries are central players in the global research landscape on stochastic models for disease prediction, likely due to their strong academic institutions, funding resources, and established networks of researchers. China also plays a significant role in the network, with numerous connections to other countries, reflecting its growing influence in scientific research and international collaboration. Other countries such as Germany, Switzerland, and Italy are also notable for their strong co-authorship links, particularly with countries like the United States and the United Kingdom. This suggests that European nations are actively engaged in collaborative research in this domain, contributing to the advancement of the field through international partnerships. Additionally, countries like India, Japan, and Brazil have established connections within the network, though to a lesser extent compared to the major hubs, indicating their participation in the global research effort, albeit on a smaller scale.

The network also highlights regional clusters, such as the collaboration between Asian countries (e.g., China, India, Japan) and between European countries (e.g., Germany, Italy, Switzerland), which may reflect regional research initiatives or shared interests in specific aspects of stochastic modeling in disease prediction. Smaller nodes like New Zealand, Israel, and Norway have fewer connections, suggesting that while these countries are involved in the research, they may have more limited collaboration or are emerging players in the field. Overall, the figure underscores the importance of international collaboration in advancing research on stochastic models for disease prediction, with major hubs like the United States, the United Kingdom, and China leading the way in fostering global partnerships. The interconnected nature of the network suggests that research in this area benefits from a diverse range of perspectives and expertise, contributing to the development of robust and innovative solutions to complex health challenges.

Co-occurrence of Keywords

**Figure 7:**
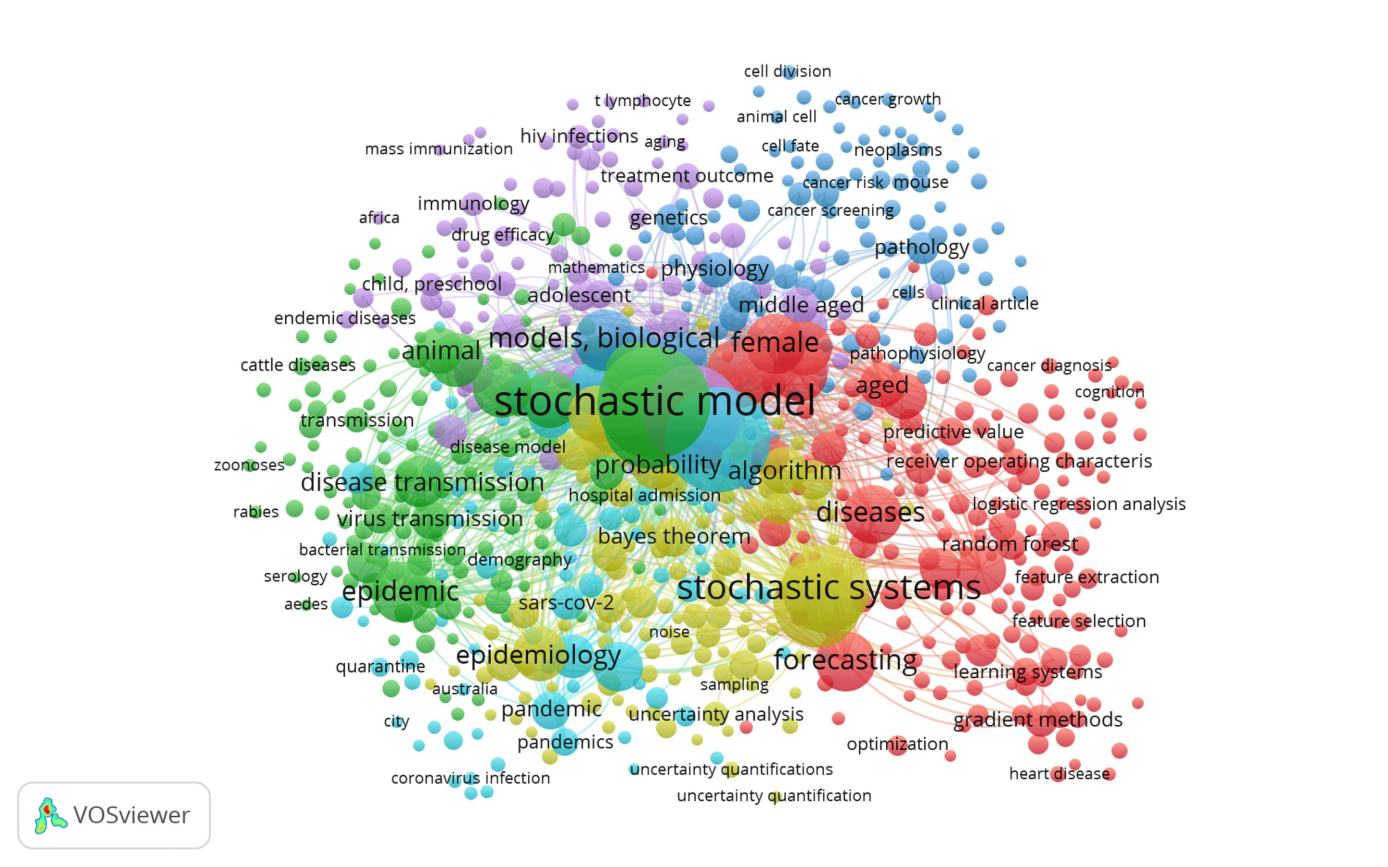
Co-occurrence of keywords

Figure 7 visualizes the co-occurrence of keywords in research related to stochastic models for disease prediction. The network map highlights the relationships between various research terms, with the size of each node representing the frequency of keyword usage and the proximity of nodes indicating how often the terms are used together in the literature.

The central and most prominent keyword is "stochastic model," indicating its pivotal role in this research area. Closely linked to it are terms like "stochastic systems," "epidemiology," "disease transmission," "forecasting," and "epidemic," which form the core of research topics in this field. These clusters demonstrate the focus on modeling and predicting disease spread, particularly in the context of epidemics and pandemics. Other significant clusters include topics related to "machine learning," "random forest," and "logistic regression analysis," reflecting the integration of advanced computational methods in analyzing and predicting disease outcomes. This integration underscores the interdisciplinary nature of the research, where computational techniques are increasingly employed alongside traditional epidemiological models.

The map also reveals a strong focus on specific disease-related terms, such as "cancer," "HIV infections," and "SARS-CoV-2," indicating the application of stochastic models in understanding and predicting the progression and transmission of these diseases. Additionally, keywords related to "immunology," "genetics," and "physiology" suggest that stochastic models are being utilized to explore biological processes at multiple levels, from cellular mechanisms to population dynamics. Overall, Figure 7 provides a comprehensive overview of the research landscape, showing how stochastic modeling intersects with various disciplines and applications, particularly in the context of disease prediction and management. The co-occurrence of keywords highlights the collaborative nature of this research field, where methods from computational sciences are combined with biological and medical sciences to address complex health challenges.

Discussion

Our results in the bibliometric analysis give rich insight into the evolution of stochastic models in disease prediction research and their current status. Among others, they indicate that since 2014, the number of publications has increased; this probably means that stochastic models are increasingly accepted as an essential tool for epidemiological studies and relevant to modern health challenges. This interest surge can be attributed to many factors, such as improved computational power, rising complexity of health data, and the dire need for predicting and managing emerging diseases like COVID-19.

Another major point arising from the analysis is the diversification of research topics over time. Initially, it was dominated by traditional applications of stochastic models in domains like HIV and cancer. However, with trend topics and co-occurrence of keywords, it clearly shows that a shift happened in stochastic modeling by incorporating more advanced computational techniques like machine learning and deep learning. This is most evident in terms of the recent global health crises, whereby accurate and timely predictions mean effective intervention strategies.

This thematic map further underlines the maturity of some research areas, with such themes as stochastic processes and epidemic modeling being both central and well-developed. These areas have contributed a great deal toward building up our insight into the dynamics of diseases, along with developing robust predictive models. In contrast, underlining themes such as gradient methods and stochastic gradient descent indicate that there is a continuous innovation in the use of these models in handling large datasets and model improvement, especially.

The co-authorship analysis brings out the global nature of research in this field, with huge contributions from the United States, the United Kingdom, and China. These countries do not only produce a high volume of research; they are also involved in deep international collaboration, mindful of the fact that this helps to move the field ahead. It would then suggest that research initiatives and funding opportunities in those regions are therefore making collaborations possible and stimulating innovation in stochastic modeling, specifically due to the fact that there are regional clusters, especially in Europe and Asia.

Although some sunny spots in the progress have been noted, there are still certain research gaps and challenges. For instance, while stochastic models are applied to a wide range of diseases, more studies on their application to low-resource settings and less-explored diseases will be greatly useful. This is another area that is yet to be fully explored: the merging of stochastic models with other predictive tools, like deterministic or agent-based models. The questions more complex than those would, in turn, be answered by piecing together the added strengths of different approaches to modeling.

Another challenge in the application of stochastic models to different studies is standardization. According to the analysis, there is a wide array of methods and models in use, which may bring about problems when trying to compare results between studies. In this respect, developing standardized protocols for model selection, validation, and reporting could improve reproducibility and comparability in this kind of research. Furthermore, the minor decrease in the number of publications from 2022 might be a change in focus or simply stabilization. Monitoring the trends in future research should be done to see whether this is truly a fluctuation or a permanent change in priorities.

A key finding of this study is the influence of regional differences and resource access on research trends and collaborations in stochastic models for disease prediction. High-resource countries like the United States, the United Kingdom, and China dominate the research landscape, driven by strong funding, access to advanced computational tools, and well-established academic infrastructures. In contrast, low-resource settings, particularly in regions such as Africa, South Asia, and Latin America, are underrepresented in collaborative networks, limiting their participation in global research efforts. This underrepresentation may lead to a lack of focus on diseases disproportionately affecting these regions as well as challenges in adapting models developed in high-resource settings to local contexts. Addressing this imbalance requires fostering greater international collaboration and capacity-building initiatives that provide researchers in low-resource settings with access to data, computational resources, and funding opportunities. Such efforts would enhance the applicability of stochastic models to a wider range of global health challenges and improve the inclusivity and impact of future research.

This bibliometric analysis gives the general trend of academic scholarship in stochastic models for disease prediction. It defines the fast-changing nature of this domain, standing out with its increasing integration of advanced computational techniques and international collaboration. In the near future, concentrated effort should be channeled into the identified gaps in research and standardization of methodologies that are key to further applications of the stochastic disease models in disease prediction to bring improvement in public health across the world.

Research Gaps

The literature reveals impressive progress in the application of stochastic models toward disease prediction, but still with some noticeable gaps. One major concern is the limited research into their application in low-resource settings. Most studies are concentrated in high-income countries where huge sets of data and better computational tools, necessary for accomplishing such tasks, can be found. This creates a gap in the understanding of how stochastic models should be appropriately adapted and applied in resource-constrained regions, where generally data can be quite sparse and/or unreliable. The developed models, due to this fact, lack generalizability across different global contexts, particularly in areas most vulnerable to outbreaks of diseases or health challenges.

Another critical gap is that very few of the published studies have analyzed integrating stochastic models with other approaches to prediction, such as deterministic and agent-based models; each modeling approach has its advantages, but few studies investigate whether there are any gains to be derived from making predictions by combining them. Such complex health scenarios would involve the interaction of many different factors, in which case it may be true that one approach alone is not good enough to produce robust and accurate predictions. Thirdly, there is also a demand for more non-communicable disease-oriented research since they emerge as leading causes of morbidity and mortality in the currently conducted studies across the world. The literature available is mostly biased toward infectious diseases, thereby leaving a wide gap in applying stochastic models to the growing burden of non-communicable diseases. The variability across studies in terms of methodologies and approaches itself goes on to reflect a lack of standardization in model development, validation, and reporting that has eventually made it quite inviable and time-consuming to compare and reproduce the claimed research findings consistently.

Practical Implications and Limitations

Key practical implications of the major insights from this study are very useful for the work of scholars, policymakers, and public health practitioners. For policymakers, the growing family of stochastic models research provides unequivocal evidence that it is high time to integrate these models into public health strategies and decision-making processes. Importantly, stochastic models account for uncertainties and variabilities that are inherent in the system and that policymakers can understand to design more flexible and robust health interventions in the event of outbreaks or for managing burdens of chronic diseases. For the researcher, the trends and influential studies identified in this field can be developed by guiding future research efforts, particularly in the less-represented areas of non-communicable diseases and low-resource settings. New computational techniques, such as machine learning integrated with stochastic models, present a range of opportunities to innovate in the handling of large, complex data sets.

From the viewpoint of public health, using stochastic models in actual disease prediction would go a long way in informing more effective and timely interventions. For example, prediction of the course of an epidemic through a stochastic model would help public health officials to allocate resources in a more efficient manner and apply focused control measures. In the chronic disease landscape, these models can further predict disease progression at levels of both the individual and population, toward the development of more tailor-fitted and effective treatment schemes. This also underlines once more the significance of international collaboration in myriad research projects. These collaborations allow for the sharing of data, resources, and expertise across borders, ultimately expanding into globally comprehensive findings of research and relevant outcomes of public health or global health.

While this study provides a comprehensive bibliometric analysis of stochastic models in disease prediction, several limitations must be acknowledged. This study relies solely on Scopus as the data source, which may have excluded relevant studies from other databases, potentially limiting the comprehensiveness of the findings. Additionally, the bibliometric analysis is largely quantitative, focusing on publication counts, citations, and co-authorship networks, which may not fully capture the qualitative aspects of the research contributions, such as the depth or real-world impact of the studies.

## Conclusions

This study provides a detailed bibliometric analysis of the scholarly landscape on stochastic models in disease prediction, highlighting significant progress over the past three decades. The recent surge in publications underscores the growing recognition of stochastic models as essential tools for addressing the complexities and uncertainties inherent in disease dynamics. The integration of advanced computational techniques, such as machine learning, with traditional stochastic modeling has further enriched the field, enabling more accurate and nuanced predictions across various health contexts, including both infectious and non-communicable diseases.

However, key research gaps persist. Notably, the application of stochastic models in low-resource settings remains underexplored, and there is a pressing need for greater standardization across studies. To improve the comparability and reproducibility of research in this field, future studies should adopt standardized methodologies for model selection, validation, and reporting. Guidelines could include clearly defined criteria for data inclusion, a standardized approach to handling uncertainties in modeling, and consistent metrics for evaluating model performance. Additionally, there is a need to ensure that models are validated in diverse contexts, particularly low-resource settings, to enhance their global applicability. Interdisciplinary collaboration is also crucial for the future of stochastic modeling. As health challenges grow more complex, integrating expertise from fields such as computational science, epidemiology, public health, and data science will be essential for developing more robust and adaptable models. Collaborative efforts should be encouraged through increased funding for interdisciplinary research initiatives and platforms that facilitate data sharing and knowledge exchange across borders.

In conclusion, stochastic models will play a central role in guiding interventions and improving disease outcomes in the coming years, particularly as the global health landscape evolves with rising pandemics and chronic diseases. By addressing the current gaps and fostering interdisciplinary collaboration, the field can continue to grow and make a substantial impact on global public health.
